# Selective restudy can reset recall of forgotten information

**DOI:** 10.3758/s13423-022-02131-y

**Published:** 2022-06-17

**Authors:** Lukas Trißl, Karl-Heinz T. Bäuml

**Affiliations:** Department of Experimental Psychology, Regensburg University, 93040 Regensburg, Germany

**Keywords:** Episodic memory, Forgetting, Restudy, Context

## Abstract

Recall of studied material is typically impaired as time between study and test increases. Selective restudy can interrupt such time-dependent forgetting by enhancing recall not only of the restudied but also of the not restudied material. In two experiments, we examined whether this interruption of time-dependent forgetting reflects a transient or more lasting effect on recall performance. We analyzed time-dependent forgetting of studied items right after study and after time-lagged selective restudy. Restudy boosted recall of the not restudied items up to the levels observed directly after study and created a restart of time-dependent forgetting from this enhanced recall level. Critically, the forgetting after restudy was indistinguishable from the forgetting after study, suggesting that restudy induced a reset of recall for the not restudied items. The results are consistent with the idea that restudy reactivates the temporal context during study, thus facilitating recall of the not restudied items. In particular, the findings suggest that such context updating reflects a lasting effect that entails a restart of the original time-dependent forgetting. Results are discussed with respect to recent, similar findings on effects of time-lagged selective retrieval.

## Introduction

Memory research has identified a wealth of methods of how to improve recall of study material (see Baddeley, Eysenck, & Anderson, 2015). Among these methods is simple restudy of the encoded material, which - though by far not the most effective method - reliably enhances recall of the material relative to a condition in which such reexposure is absent. The effect has typically been demonstrated when all of the encoded material was subject to reexposure (see Crowder, 1976, Chap. 9), but the finding also generalizes to a situation in which restudy is selective and a subset of the material is reexposed for restudy only. In such case, the effect of restudy is indeed selective: recall of the restudied items is improved, whereas recall of the not restudied items is left unaffected (Bäuml & Aslan, 2004; Ciranni & Shimamura, 1999; Verde, 2013).

A feature shared by most studies on selective restudy is that restudy followed shortly upon study of the items, with the temporal lag between study and restudy being no longer than 1 min. Against this background, a few recent studies demonstrated that the effects of selective restudy can change when the temporal lag between study and restudy is prolonged (Bäuml & Dobler, 2015; Wallner & Bäuml, 2017). Employing lags of 10 min, 30 min, or 48 h, these studies found that lagged restudy not only improves recall of the restudied items but improves recall of the not restudied items as well. Because recall typically declines as time since study increases - reflecting so-called time-dependent forgetting (Ebbinghaus, 1885; Slamecka & McElree, 1983) - the finding that time-lagged restudy can enhance recall of the not restudied material indicates that selective restudy can interrupt the material’s time-dependent forgetting.

The interruption of time-dependent forgetting as caused by selective restudy has been attributed to context retrieval (Bäuml, 2019; Wallner & Bäuml, 2017). Studied items are linked to the temporal context in which they are shown (Howard & Kahana, 2002; Raaijmakers & Shiffrin, 1981), but context changes over time (Bower, 1972; Estes, 1955). Such change makes context immediately before lagged restudy starts different from context during study, which can impair recall performance. Reexposure of a studied item, however, can reactivate the context that was present when that item was studied, and this retrieved context then updates current temporal context, which can serve as a retrieval cue for other items with a similar context at study (Howard & Kahana, 2002; Polyn & Kahana, 2008). Thus, if restudied and not restudied items share contextual features encoded during study, lagged selective restudy can reactivate part of the study context of the not restudied items, facilitate recall of these items, and interrupt the items’ time-dependent forgetting.

While there is thus evidence that selective restudy can interrupt time-dependent forgetting, it is still unclear whether this interruption reflects a short-lived or a more lasting effect on recall performance. Arguably, the context updating induced by context retrieval may reflect a transient discontinuity in the stream of temporal context only (Folkerts, Rutishauser, & Howard, 2018), with study context remaining available for a short time after restudy but recall quickly falling back to the trajectory of forgetting it was already on. Alternatively, the induced context retrieval may cause a lasting updating effect. It may effectively shift the study context closer to current temporal context (Lohnas, Polyn, & Kahana, 2011), which may reset the recall process and induce a restart of time-dependent forgetting. Such a pattern has recently been reported in a study on selective retrieval, in which time-lagged selective retrieval was found to enhance recall of the nonretrieved material and, from this enhanced recall level, induce a restart of time-dependent forgetting (Bäuml & Trißl, 2022). However, the effects of selective restudy and selective retrieval have repeatedly been shown to differ in detail (see Bäuml & Kliegl, 2017) and to also differ in degree of induced study context reactivation (Bäuml & Dobler, 2015; Wallner & Bäuml, 2017). Whether selective restudy parallels selective retrieval and induces lasting context updating therefore remains unclear.

This study reports the results of two experiments designed to examine how lagged selective restudy influences time-dependent forgetting of the not restudied material. In each experiment, time-dependent forgetting of studied items when recall was tested after study in the absence of selective restudy was compared with time-dependent forgetting of restudied and not restudied items when recall was tested after selective restudy. During selective restudy, some studied items were reexposed, thus creating restudied and not restudied items. In Experiment 1, selective restudy occurred 10 min after study, in Experiment 2 it occurred 30 min after study; both lag intervals typically induce time-dependent forgetting (Kliegl, Carls, & Bäuml, 2019; Wallner & Bäuml, 2017). Both when recall was tested after study and when it was tested after selective restudy, recall was assessed at different delay intervals, which allowed a comparison of the time-dependent forgetting with and without preceding selective restudy. We expected that restudy induced an initial recall boost for the not restudied items directly after restudy, thus interrupting the items’ time-dependent forgetting (Wallner & Bäuml, 2017). Critically, we expected the results to inform us on whether the induced interruption of time-dependent forgetting is a transient or a more lasting phenomenon. If lasting, the time-dependent forgetting of the not restudied items after selective restudy may mimic the time-dependent forgetting directly after study, suggesting that restudy induced a reset of the recall process for these items and a restart of time-dependent forgetting.

## Experiment 1

### Method

#### Ethical considerations

All reported studies were carried out in accordance with the provisions of the World Medical Association Declaration of Helsinki.

##### Participants

A total of 192 students of Regensburg University took part in the experiment (*M* = 22.56 years, range 18–32 years, 81.8% females). They were equally distributed across the eight between-participants conditions, resulting in 24 participants in each condition. We determined the desired sample size guided by the results of a power analysis (Faul, Erdfelder, Lang, & Buchner, 2007) using alpha = 0.05 and beta = 0.20 as well as an effect size of d = 0.80 for expected time-dependent forgetting and expected beneficial effects of selective restudy (Kliegl et al., 2019; Wallner & Bäuml, 2017). All subjects provided informed consent and were tested individually in the laboratory. They received monetary reward or course credit for participation.

##### Materials

A list of 15 unrelated concrete German nouns served as study material, taken from the prior study by (Wallner & Bäuml, 2017). Each item had a unique initial letter. The items are referred to as studied items when selective restudy was absent. When selective restudy was present, the restudied items are referred to as practiced items and the not restudied items as unpracticed items. In such case, ten items of the list served as the practiced items and the other five items served as the unpracticed items. For this, the list was divided into three sets of five items each. Each set served equally often as practiced items and equally often as unpracticed items within each restudy condition.

##### Design and procedure

**Fig. 1:**
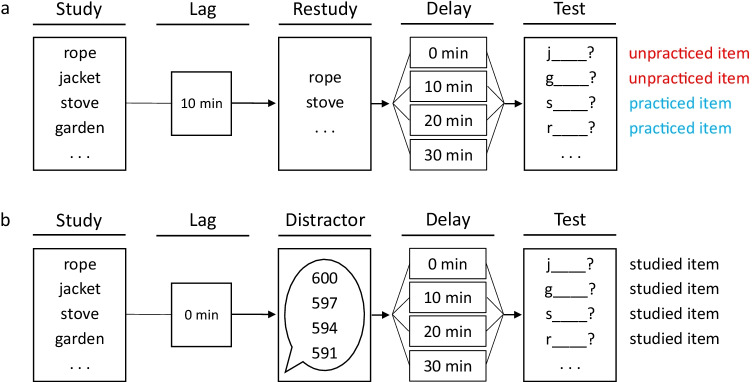
Experimental design for Experiment 1. (**a**) Recall was tested after selective restudy, which occurred 10 min after study and created practiced and unpracticed items. Between restudy and test, there were delays of 0, 10, 20, or 30 min. (**b**) Recall was tested after study and a subsequent distractor task in the absence of selective restudy. Between distractor and test, there were delays of 0, 10, 20, or 30 min

One half of the participants engaged in selective restudy 10 min after study, whereas the other half of the participants engaged in an unrelated distractor task of the same duration as the restudy period directly after study (see Fig. 1). Across participants, delay intervals of 0, 10, 20, or 30 min were employed between restudy and test. When restudy was absent, the same four delay intervals were employed between distractor and test.

In the study phase, the items of the list were exposed individually on a computer screen for 6 s each and in a random order. The lag of 10 min between study and selective restudy as well as the delay intervals of 10, 20, and 30 min between selective restudy and test and between distractor and test were filled with blocks of neutral distractor tasks. Each block lasted for 10 min and consisted of three different tasks: the first task involved the connect-the-numbers test (Oswald & Roth, 1987), rating pictures of places, mental arithmetics, or the d2 test of attention (Brickenkamp & Zillmer, 1998); the second task involved playing Tetris, solving decision tasks, standard progressive matrices (Raven, Raven, & Court, 2000), or ordering number triplets; and the third task involved a mental imagination task, in which participants were asked to think back to an international vacation, imagine winning the lottery, imagine being invisible, or imagine walking through the childhood home (Delaney, Sahakyan, Kelley, & Zimmerman, 2010). For each of the three tasks of a block, the single distractors were sampled randomly from the task’s set of possible four distractors. Across blocks, the sampling was conducted without replacement.

During selective restudy, 10 of the 15 initially studied items (i.e., the practiced items) were reexposed in random order. Participants were asked to study the items a second time, for 6 s each. There were two successive practice cycles. When restudy was absent, participants counted backwards for the equivalent time interval of 2 min immediately after study. At test, all participants were asked to recall all 15 items. Order of tested items was random but in the selective restudy conditions, the unpracticed items were always tested first and the practiced items last (see Wallner & Bäuml, 2017). Participants had 6 s to recall a single item. The items’ first letter served as a retrieval cue for recall of the corresponding item. Responses were given orally.

##### Fitting the power function to the recall rates

Following prior work on time-dependent forgetting (Bäuml & Trißl, 2022; Rubin & Wenzel, 1996; Wixted & Ebbesen, 1991, 1997), we fitted a power function of time, *r*(*t*) = *at*^−*b*^, to the recall rates of the studied, practiced, and unpracticed items. In this function, *r*(*t*) represents percentage of recalled items at time *t*, parameter *b* represents the forgetting rate as time increases, and parameter *a* represents recall level after one unit of time (i.e., 1 min after study for the studied items and 1 min after selective restudy for the practiced and unpracticed items). The fitting procedure followed the one employed in Bäuml and Trißl (2022).

The power function was fitted to the recall rates using maximum likelihood methods (Riefer & Batchelder, 1988; Wickens, 1982). The functions were fit to group average data (Wixted & Ebbesen, 1997). The goodness-of-fit of the power function was compared to the goodness-of-fit of a statistical baseline model, which describes the recall rates of an item type - practiced, unpracticed, or studied items - for *n* delay conditions as the product of *n* independent binomial distributions. The comparison of the power function versus the statistical baseline model is based on the calculation of a likelihood ratio and leads to an approximative *χ*^2^-test with *n* − 2 degrees of freedom (Riefer & Batchelder, 1988; Wickens, 1982). Estimation of the parameters of the power function was achieved by maximizing the likelihood ratio.^1^

Next, for each pair of item types, it was examined whether parameters *a* and *b* of the power function varied with item type. For this analysis, the data sets of the two item types were combined and the goodness-of-fit of the general power function model - which allows separate power functions for the two item types and thus includes two free *a*-parameters and two free *b*-parameters - was compared with a restricted power function model on which the restriction of a common parameter *a* was imposed (e.g., Riefer & Batchelder, 1988). Again, the comparison between the two models was based on calculation of a likelihood ratio and a *χ*^2^ test with one degree of freedom. Analogously, it was examined whether parameter *b* varied with item type. Notice that if parameter *a* did not vary between two item types, the restriction of a common parameter *a* was imposed on both the general and the restricted power function model for the test of the constancy of parameter *b* (e.g., Riefer & Batchelder, 1988). With such a procedure, a stronger statistical test of the constancy of parameter *b* is possible than is the case when the two *a* parameters are allowed to vary freely.

## Results

Typical time-dependent forgetting emerged when testing occurred directly after study in the absence of selective restudy (Fig. 2a). Consistently, an unifactorial analysis of variance (ANOVA) with the between-subjects factor of delay (0 min vs. 10 min vs. 20 min vs. 30 min) indicated that recall of the studied items decreased as the delay interval between study and test increased, *F*(3,92) = 4.51, *MSE* = 0.03, *p* = .005, *η*^2^ = 0.13.^2^ However, selective restudy interrupted this forgetting. Recall of the practiced items right after selective restudy was enhanced relative to recall of the studied items when these items were tested after the same time interval since study (practiced items versus studied items: *M* = 87.08%, 95% *CI* = [81.31, 92.85] versus *M* = 51.67%, 95% *CI* = [43.61, 59.73]), *t*(46) = 7.39, *p* < .001, *d* = 2.13. An analogous recall enhancement was present for the unpracticed items (unpracticed items versus studied items: *M* = 65.83%, 95% *CI* = [57.77, 73.90] versus *M* = 51.67%), *t*(46) = 2.57, *p* = .013, *d* = 0.74, although the recall boost for the practiced items exceeded that for the unpracticed items, *t*(23) = 4.66, *p* < .001, *d* = 0.95. As time after selective restudy passed, both the practiced and the unpracticed items again showed time-dependent forgetting, with recall decreasing as the delay interval between restudy and test increased. A corresponding ANOVA with the between-subjects factor of delay (0 min vs. 10 min vs. 20 min vs. 30 min) and the within-subject factor of item type (practiced vs. unpracticed items) revealed main effects of delay, *F*(3,92) = 3.10, *MSE* = 0.05, *p* = .031, *η*^2^ = 0.09, and item type, *F*(1,92) = 74.25, *MSE* = 0.03, *p* < .001, *η*^2^ = 0.45, but no interaction between the two factors, *F*(3,92) < 1.

To quantify time-dependent forgetting, we fitted the power function, *r*(*t*) = *at*^−*b*^, to the recall rates of the studied, practiced, and unpracticed items (Fig. 2a). The function described time-dependent forgetting of the three item types well, as is reflected by the *χ*^2^(2) values of 0.71 for the studied items, 1.80 for the practiced items, and 0.03 for the unpracticed items. We analyzed whether the function’s two parameters varied between item types. Regarding studied and unpracticed items, the two item types did not differ in parameter *a*, *χ*^2^(1) = 2.38, and also did not differ in parameter *b*, *χ*^2^(1) = 1.56, indicating that time-dependent forgetting directly after study in the absence of selective restudy and time-dependent forgetting of unpracticed items after selective restudy were comparable. Regarding studied and practiced items, a different picture arose. The two item types differed both with respect to parameter *a*, *χ*^2^(1) = 10.20, and with respect to parameter *b*, *χ*^2^(1) = 11.02, with a larger parameter *a* and a smaller parameter *b* for the practiced items, indicating that time-dependent forgetting was reduced for the practiced items. Practiced and unpracticed items differed in parameter *a*, *χ*^2^(1) = 39.30, but did not differ in parameter *b*, *χ*^2^(1) = 1.68.

## Discussion

In line with prior work, the results of the experiment show typical time-dependent forgetting of studied items (Rubin & Wenzel, 1996; Wixted & Ebbesen, 1991), and they demonstrate that selective restudy 10 min after study can interrupt such time-dependent forgetting, for both the practiced and the unpracticed items (Wallner & Bäuml, 2017). The recall level of the unpracticed items directly after restudy was similar to the recall level of the studied items directly after study, suggesting that restudy eliminated the time-dependent forgetting that had occurred since study. Moreover, from this enhanced recall level, the time-dependent forgetting of the unpracticed items after restudy mimicked the time-dependent forgetting of the studied items, indicating that the interruption of time-dependent forgetting for the unpracticed items was not a transient phenomenon and restudy rather induced a reset of recall for these items and a restart of time-dependent forgetting. The recall level of the practiced items directly after restudy was superior to the recall levels of the other item types and the practiced items even showed reduced time-dependent forgetting relative to the studied items, suggesting that restudy can attenuate the forgetting for practiced items. The goal of Experiment 2 was to replicate the results of Experiment 1 using a longer lag of 30 min between study and selective restudy and employing other study material. Besides, Experiment 2 was highly similar to Experiment 1.

## Experiment 2

### Method

#### Participants

A total of 192 students (*M* = 24.1, range 18–34 years, 83.9% females) of different German universities participated in the experiment and were tested individually in an online video conference hosted by the software Zoom (Zoom Video Communications Inc., 2016). The experimenter provided the instructions and was present for the entire period of the experiment. Sample size followed Experiment 1. The participants were equally distributed across the eight between-participants conditions, resulting in 24 participants in each condition.

#### Materials

Another list of 15 unrelated concrete German nouns served as study material, again taken from the prior study by Wallner and Bäuml (2017). Each item had a unique initial letter. The division of the items into studied, practiced, and unpracticed items followed Experiment 1.

#### Design and procedure

Design and procedure were largely identical to Experiment 1 but differed in three aspects from the preceding experiment: (a) selective restudy occurred 30 min after study; (b) in the selective restudy conditions, a 2-min counting task was introduced directly after selective restudy, so that the procedure after selective restudy was identical to the procedure after study; (c) the delay intervals of 10 min, 20 min, and 30 min as well as the lag of 30 min between study and selective restudy were again filled with neutral distractor tasks; in contrast to Experiment 1, however, each 10-min interval now contained a single distractor task only; in each condition, the distractor tasks were selected randomly (without replacement) from a set of six different distractor tasks: spot-the-difference puzzles, mental arithmetics, standard progressive matrices, operation span task (Turner & Engle, 1989), brain teasers, and decision tasks.

#### Results

Like in Experiment 1, typical time-dependent forgetting emerged when testing occurred directly after study in the absence of selective restudy (Fig. 2b). A unifactorial ANOVA with the between-subjects factor of delay (0 min vs. 10 min vs. 20 min vs. 30 min) indeed indicated a main effect of delay interval, *F*(3,92) = 5.18, *MSE* = 0.03, *p* = .002, *η*^2^ = 0.14. Selective restudy interrupted this forgetting. Recall of both the practiced and the unpracticed items were boosted right after selective restudy relative to recall of the studied items when these items were tested after about the same time interval since study (practiced items versus studied items: *M* = 80.00%, 95% *CI* = [74.57, 85.43] versus *M* = 46.67%, 95% *CI* = [38.37, 54.97], *t*(46) = 6.95, *p* < .001, *d* = 2.01; unpracticed items versus studied items: *M* = 64.17%, 95% *CI* = [54.53, 73.81] versus *M* = 46.67%, *t*(46) = 2.85, *p* = .007, *d* = 0.82).^3^ Again, recall of the practiced items was more enhanced by restudy than was recall of the unpracticed items, *t*(23) = 3.55, *p* = .002, *d* = 0.72. As time after restudy increased, both the practiced and the unpracticed items were again susceptible to time-dependent forgetting. An ANOVA with the between-subjects factor of delay (0 min vs. 10 min vs. 20 min vs. 30 min), and the within-subject factor of item type (practiced vs. unpracticed items) showed a marginally significant main effect of delay, *F*(3,92) = 2.51, *MSE* = 0.05, *p* = .063, *η*^2^ = 0.08, and a main effect of item type, *F*(1,92) = 66.48, *MSE* = 0.03, *p* < .001, *η*^2^ = 0.42, but no interaction between the two factors, *F*(3,92) < 1.

To quantify time-dependent forgetting, we again fitted the power function, *r*(*t*) = *at*^−*b*^, to the recall rates of the studied, practiced, and unpracticed items (Fig. 2b). The power function described the recall rates of the three item types well, with *χ*^2^(2) values of 0.50 for the studied items, 3.42 for the practiced items, and 0.89 for the unpracticed items. Like in Experiment 1, studied and unpracticed items did not differ in the function’s parameter *a*, *χ*^2^(1) = 0.03, and did also not differ in the function’s parameter *b*, *χ*^2^(1) = 1.49, suggesting that time-dependent forgetting of unpracticed items after selective restudy was indistinguishable from time-dependent forgetting right after study. Results were different for the comparison between studied and practiced items. The two item types differed with respect to both parameter *a*, *χ*^2^(1) = 5.22, and parameter *b*, *χ*^2^(1) = 8.14, with a larger parameter *a* and a smaller parameter *b* for the practiced items, indicating that time-dependent forgetting was reduced for the practiced items. Practiced and unpracticed items did not differ in parameter *a*, *χ*^2^(1) = 3.28, but differed in parameter *b*, *χ*^2^(1) = 57.46, again suggesting reduced forgetting for the practiced items.^4^

## Discussion

The results largely replicate those of Experiment 1. They show time-dependent forgetting for the studied items and a recall boost for both the practiced and the unpracticed items right after selective restudy. Again, the recall level of the unpracticed items directly after restudy was similar to the recall level of the studied items directly after study, whereas the recall level of the practiced items exceeded the recall level of the other two item types. Critically, time-dependent forgetting of the unpracticed items after restudy again mimicked time-dependent forgetting of the studied items in the absence of restudy, which indicates that the interruption of time-dependent forgetting of the unpracticed items was accompanied by a restart of the forgetting. In contrast, time-dependent forgetting of the practiced items after restudy was again reduced relative to the time-dependent forgetting of the studied items, suggesting that, from the enhanced recall level, restudy can attenuate the forgetting of the practiced items.

## General discussion

The results of the two experiments replicate prior work by showing that selective restudy can interrupt time-dependent forgetting by inducing a recall boost for the not restudied information (Bäuml & Dobler, 2015; Wallner & Bäuml, 2017). Going beyond the prior work, the results demonstrate that, on this enhanced recall level, the not restudied information reveals subsequent time-dependent forgetting that mimics time-dependent forgetting directly after study. The finding indicates that the initial recall boost for the not restudied information does not just reflect a transient discontinuity in the stream of temporal context, but selective restudy creates a reset of recall for this information and a restart of time-dependent forgetting.

As expected, selective restudy did also enhance recall of the restudied information, and this enhancement was even larger than that of the not restudied information. Moreover, the restudied information showed reduced time-dependent forgetting relative to the studied items, indicating that restudy can slow the forgetting process for the restudied information. Given that time-dependent forgetting of the not restudied items was similar to time-dependent forgetting of the studied items, and time-dependent forgetting of the restudied items was reduced relative to that of the studied items, ideally, time-dependent forgetting of the restudied items should also turn out to be reduced relative to the not restudied items. Such pattern in fact emerged in Experiment 2, whereas in Experiment 1 it was present numerically but not statistically. Further studies, preferably with increased statistical power, should therefore bolster the suggestion that restudied items also show attenuated forgetting relative to the not restudied items.

The findings of this study are consistent with the proposal that selective restudy induces context retrieval that updates context by adding the restudied items’ study context to the current state of temporal context (Bäuml, 2019). Critically, the findings also indicate that such updating reflects a lasting effect, entailing a restart of time-dependent forgetting for the not restudied items that parallels the one for studied items directly after study. For the restudied information, the results also suggest a restart of time-dependent forgetting, although in this case the forgetting is reduced relative to the studied items. This finding indicates that, with regard to the restudied items themselves, restudy did more than reactivating the study context. It may, for instance, have induced a more elaborate processing of the restudied items or a higher level of memory consolidation relative to the studied items, both of which might reduce forgetting over time (Carpenter, 2009; Wixted, 2004). However, while it is not entirely clear how elaborative processing would slow time-dependent forgetting, enhanced consolidation may attenuate forgetting by gradually transforming newly encoded information into more and more stable memory representations (Dudai, Karni, & Born, 2015; Wixted, 2004).

In a recent study, Bäuml and Trißl (2022) reported that lagged selective retrieval improved recall of both the retrieved and the nonretrieved items, and improved recall of the two types of items to a similar degree. In particular, time-dependent forgetting of the nonretrieved items after selective retrieval paralleled time-dependent forgetting of studied items directly after study, whereas the retrieved items did hardly show any time-dependent forgetting. A comparison of results with the present study suggests that lagged selective retrieval and lagged selective restudy may show similar effects on the unpracticed items - and thus be similar in study context reactivation - but differ in effects on the practiced items. Indeed, selective restudy may induce a higher initial recall boost than retrieval for the practiced items, whereas selective retrieval may not only reduce but nearly eliminate the time-dependent forgetting of these items, which mimics findings from the testing effect literature (Kornell, Bjork, & Garcia, 2011; Roediger & Karpicke, 2006). Future studies may examine the suggestion by comparing the effects of the two forms of selective item repetition directly.

The experimental task used here as well as the experimental task employed in prior work on effects of time-lagged selective retrieval (Bäuml & Trißl, 2022; Wallner & Bäuml, 2017) contain some specific features. For instance, during practice, two-thirds of the studied material are practiced and practice is conducted in two successive practice cycles, features that are likely to create relatively high chances for context reactivation; or, at test, item-specific retrieval cues are presented and the unpracticed items are tested before the practiced items, features that permit rather direct measurement of unpracticed items’ context reactivation. Probably, the observed benefits of practice would decrease only slightly if a smaller proportion of the studied material was practiced or a single practice cycle was conducted only. Whether testing the practiced items first and the unpracticed items last or an alternative free recall format - in which the (stronger) practiced items would also tend to be recalled first - would influence results is less clear. Prior recall of the practiced items could serve as an additional opportunity for context reactivation and thus potentially benefit the unpracticed items, but it could also impair recall of the unpracticed items if retrieval competition between items arose and recall of the practiced items blocked or inhibited recall of the unpracticed items. Examining the possible role of test format in the present experimental task looks like an interesting research project.

## Conclusions

Selective restudy provides an efficient way to not only interrupt time-dependent forgetting of the restudied material but to interrupt the forgetting of the not restudied information as well. Moreover, selective restudy creates a reset of recall of the not restudied items and thus induces a restart of the items’ time-dependent forgetting. These findings are of relevance for both memory theory and application, for instance, educational settings, where knowing that selective restudy can be sufficient to revive also the not restudied information can be highly beneficial. Generalization of the present results to more applied settings may therefore be a high priority for future work.

## Footnotes

1 The parameters of the power function were also estimated by using nonlinear regression analysis (Rubin & Wenzel, 1996; Wixted & Ebbesen, 1991). Results turned out to be indistinguishable between methods.

2 Because participants in the selective restudy conditions recalled the five unpracticed items first and the ten practiced items last, we examined whether recall of studied items in the first five output positions differed from recall of the items in the last ten output positions. In such a case, the studied items retrieved in the first five output positions should serve as baseline for the unpracticed items, and the studied items retrieved in the last ten output positions serve as baseline for the practiced items. However, in both Experiment 1 and Experiment 2, corresponding ANOVAs revealed no main effect of output position, *p**s* > .266, and no interaction between output position and delay interval, *p**s* > .812. We therefore pooled the two sets of studied items within each of the two experiments.

3 In contrast to Experiment 1, a 2-min distractor task was included in Experiment 2 right after selective restudy (see Method above). As a result, recall of the practiced and unpracticed items right after restudy was measured 2 min later since study than recall of the corresponding studied items. If anything, this difference in delay between study and test should have underestimated the induced recall boost for the practiced and unpracticed items.

4 Arithmetically averaging recall rates over participants can produce averaging artifacts, for instance, a group function with mathematical properties that are not representative of the individual participant data (Estes, 1956; Sidman, 1952). One way to evaluate whether or not averaging artifacts were responsible for the present results is to reanalyze the data using geometric averaging (Anderson & Tweney, 1997; Wixted & Ebbesen, 1997). Geometric averaging led to the same pattern of results as arithmetical averaging: for the studied items, estimates of forgetting parameter *b* were .120 and .131 in Experiments 1 and 2, for the practiced items .045 and .041, and for the unpracticed items .071 and .121.
